# Nanoscale mapping of dielectric properties based on surface adhesion force measurements

**DOI:** 10.3762/bjnano.9.84

**Published:** 2018-03-16

**Authors:** Ying Wang, Yue Shen, Xingya Wang, Zhiwei Shen, Bin Li, Jun Hu, Yi Zhang

**Affiliations:** 1 Key Laboratory of Interfacial Physics and Technology and Laboratory of Physical Biology, Shanghai Institute of Applied Physics, Chinese Academy of Sciences, Shanghai 201800, China; 2 Key Laboratory of Salt Lake Resources Chemistry of Qinghai Province, Qinghai Institute of Salt Lakes, Chinese Academy of Sciences, Xining, Qinghai 810008, China; 3 University of Chinese Academy of Sciences, Beijing 100049, China

**Keywords:** adhesion, atomic force microscopy (AFM), graphene oxide (GO), nanoscale dielectric properties, reduced graphene oxide (RGO)

## Abstract

The detection of local dielectric properties is of great importance in a wide variety of scientific studies and applications. Here, we report a novel method for the characterization of local dielectric distributions based on surface adhesion mapping by atomic force microscopy (AFM). The two-dimensional (2D) materials graphene oxide (GO), and partially reduced graphene oxide (RGO), which have similar thicknesses but large differences in their dielectric properties, were studied as model systems. Through direct imaging of the samples with a biased AFM tip in PeakForce Quantitative Nano-Mechanics (PF-QNM) mode, the local dielectric properties of GO and RGO were revealed by mapping their surface adhesion forces. Thus, GO and RGO could be conveniently differentiated. This method provides a simple and general approach for the fast characterization of the local dielectric properties of graphene-based materials and will further facilitate their applications in energy generation and storage devices.

## Introduction

The local dielectric distribution is a key factor that influences the physical properties and functionalities of various materials such as polymer nanocomposites [1–4], carbon nanotube compounds [5–8], metal–dielectric films [9–12], and biomembranes [13–15]. Understanding the behaviour of these complex nanostructured systems requires precise morphological and dielectric characterization approaches on the nanometre scale. Atomic force microscopy (AFM), which analyses the interactions between a sharp tip and samples with very high spatial resolution, is a good candidate to carry out the aforementioned tasks. In the last two decades, many AFM-based techniques have been developed for qualitatively or quantitatively detecting the local dielectric properties of nanomaterials, such as electrostatic force microscopy [16–19], scanning polarization force microscopy (SPFM) [20–23], local dielectric spectroscopy [24–26], and nanoscale capacitance microscopy [27–29]. Most of the proposed techniques are based on long-range electrostatic interactions between the sample and a biased AFM tip, which in turn is closely related to the intrinsic dielectric properties of materials. In this regard, one of the primary disadvantages of these dielectric-related AFM measurements is their lower lateral resolution compared to the conventional AFM modes, which is attributed to the larger tip–sample distance [30]. Moreover, in ambient electrical AFM scanning, relative humidity usually has a strong impact on image resolution and contrast [31–32].

We propose that fast mapping of the local dielectric distribution on a sample surface can be achieved with high lateral resolution by combining the advantages of the electrowetting (EW) effect [33] and an AFM imaging mode, PeakForce Quantitative Nano-Mechanics (PF-QNM) [34]. Electrowetting is a phenomenon in which the wetting properties of a dielectric surface are modified using an external electric field [33]. At the nanometre scale, EW has also been observed to modify the adhesion force [35–37]. The adhesion force between an AFM tip with radius *R* and a flat surface with liquid absorbed on it can be expressed as [35–38]:

[1]
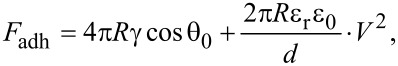


where *V* is the voltage applied on the AFM tip, γ is the liquid interfacial tension, θ_0_ is the contact angle at zero external voltage, and *d*, ε_r_ and ε_0_ are the thickness, relative permittivity of the dielectric layer, and the absolute dielectric permittivity of vacuum, respectively. Hence, the adhesion force between the AFM tip and the sample is affected by both of the wetting and dielectric properties of the sample. Based on this principle, a quantitative analysis on the dielectric constant of macroscopic film has been realized by measuring the surface–water contact angle and adhesion force between the dielectric layer and a biased AFM tip [38].

Recently, the newly-developed PF-QNM mode of AFM made it possible to simultaneously map the adhesion property as well as topography of the sample with high spatial resolution. In PF-QNM mode, force–distance curves between the AFM tip and the sample are measured at each pixel, so the force where the tip finally breaks free of the surface attraction in the withdraw direction can be extracted for adhesion mapping. This offers an opportunity to directly image the adhesion over the whole scanning area rather than only record force–distance curves at specific points on the sample.

In this letter, a method to qualitatively characterize the local dielectric distribution by adhesion mapping between a dielectric layer and a biased AFM probe is described. With this method we can simultaneously obtain the topographic and dielectric properties of the sample surface under ambient conditions without requiring reference samples [39] or lifting of the AFM tip to scan for a second time [40], which may result in a lower spatial resolution. The method was validated by local dielectric mapping of graphene oxide (GO) and reduced graphene oxide (RGO), which have similar thicknesses but large differences in their dielectric properties [21]. This approach is expected to provide a simple and convenient method to characterize the dielectric distribution of graphene-based materials, and will further facilitate their application in energy generation and storage devices, i.e., super-capacitor, lithium ion battery, solar cells, and fuel cells [41–42].

## Results and Discussion

**Figure 1:**
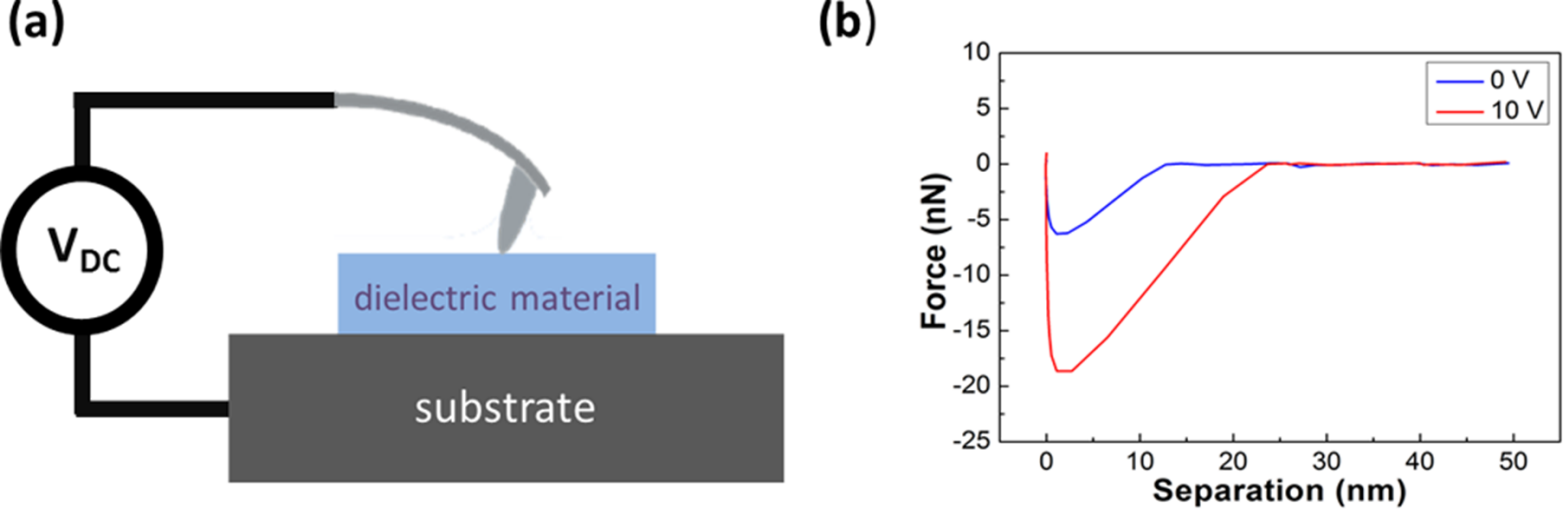
Schematic representation of the experimental principle. (a) A dc voltage can be applied to the AFM tip in PF-QNM mode under ambient conditions. (b) Representative retracting lines of force–distance curves under tip biases of 0 V and 10 V. The adhesion force between the tip and the sample will increase due to polarization of the sample when the tip is biased at 10 V.

A schematic diagram indicating the working principle of dielectric property mapping based on the adhesion force in the PF-QNM mode is shown in Figure 1. A dc voltage can be applied to the AFM tip in the PF-QNM mode under ambient conditions (Figure 1a). The force–distance curves are measured at every pixel in the scan range, and the peak forces below the baselines in the retracting line of the force–distance curves are then used for adhesion mapping. According to Equation 1, once the AFM tip is biased, the adhesion force between the tip and the sample, *F*_adh_, will increase due to sample polarization (Figure 1b), which is positively correlated to its dielectric constant. Therefore, adhesion force mapping under a biased AFM tip can be expected to characterize the local dielectric property distribution.

**Figure 2:**
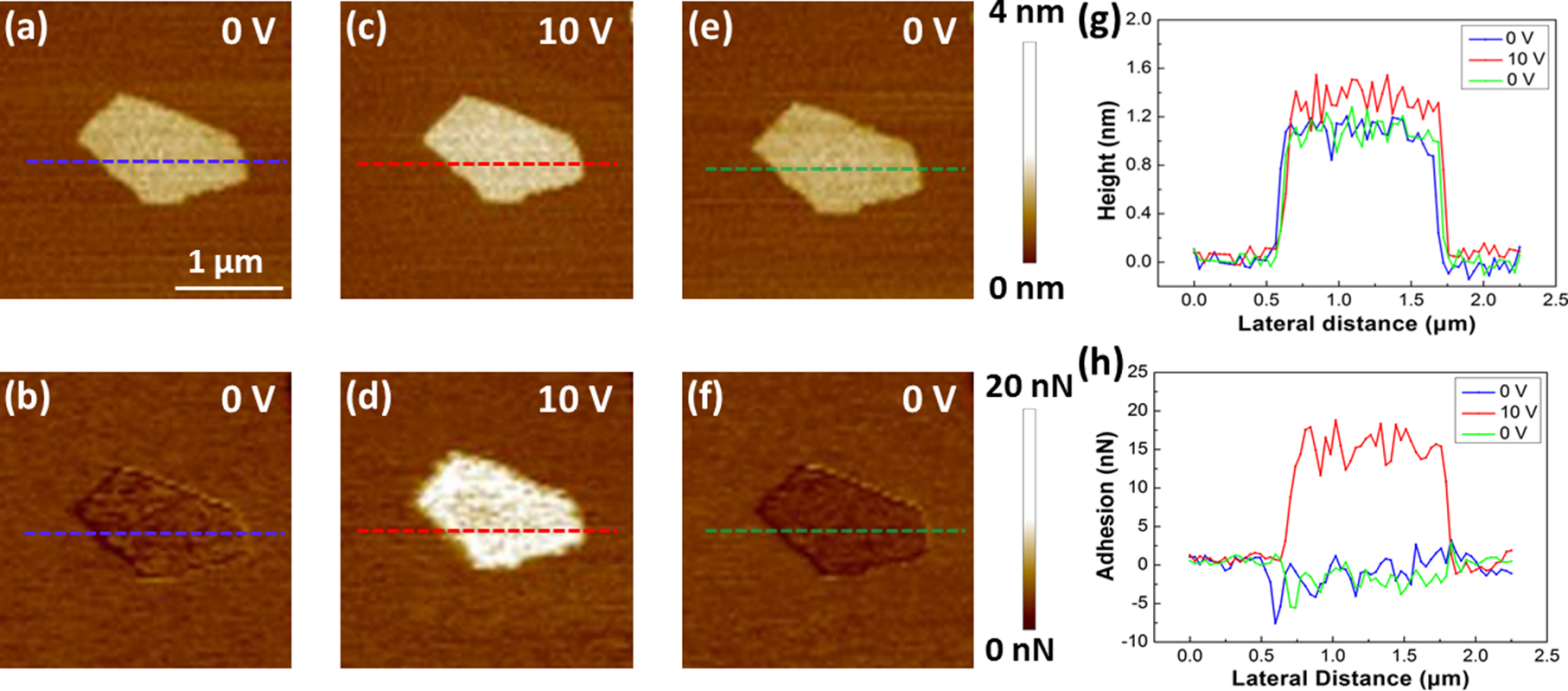
AFM height and adhesion images of single-layered CRGO sheets under different tip biases obtained in PF-QNM mode. (a,c,e) Height images obtained with an AFM tip biased at 0 V, 10 V, and 0 V, respectively. (b,d,f) Adhesion images corresponding to (a), (c), and (e), respectively. All of the images were taken with a peakforce set point of 5 nN. (g) Cross-sectional profiles along the blue, red, and green dashed lines in (a), (c), and (e), respectively. (h) Cross sectional profiles of the blue, red, and green lines in (b), (d), and (f), respectively.

An example of adhesion force mapping with a biased AFM tip in PF-QNM mode is shown in Figure 2. The height images of a single-layered GO sheet, which was chemically reduced (thus named as CRGO) by saturated hydrazine vapour on a mica surface, showed little change when the AFM tip bias increased from 0 to 10 V under ambient conditions (room temperature 18–25 °C, and relative humidity (RH) 35–60%) (Figure 2a,c). However, the contrast of the corresponding adhesion images increases significantly (Figure 2b,d). When the tip bias was lowered back to 0 V, both height and adhesion images returned to the original state before the dc voltage was applied (Figure 2e,f). Figure 2g and Figure 2h display the cross-sectional profiles along the blue, red, and green lines in Figure 2a,c,e and Figure 2b,d,f, respectively. The section profiles reveal that the change of the apparent heights was very small (ca. 0.2 nm) as the tip bias increased from 0 to 10 V and then dropped back to 0 V. Meanwhile, the adhesion force increased from −2.7 nN to 16.6 nN when the tip bias increased from 0 V to 10 V, and then returned to −2.7 nN when the tip bias was set back to zero. All measured values of the adhesion forces of the CRGO sheets are relative to that of the mica substrate. This result indicates the increase in the adhesion force when the AFM tip is biased is due to the greater degree of polarization of CRGO with respect to the mica substrate, rather than charge injection into the CRGO sheets [43]. In this case, the apparent height of the CRGO sheet under the biased AFM tip changed very little, which is quite different from the result in our previous SPFM experiment, in which the apparent height of RGO sheets under a biased tip usually increased sharply when RH was lower than 40% [32]. This is because the set point of the force, which is used as the feedback signal for AFM imaging, is quite different in PF-QNM mode and SPFM mode. Specifically, the set point of force for SPFM imaging is usually selected in the long-range attraction region of the force–distance curve, so a higher apparent height than the real value of the sample is normally observed [30]. In contrast, the set point of force for PF-QNM imaging is the peak value of the force–distance curve, which is usually in the repulsive region. Therefore, the effect of long-range attraction between a biased tip and the sample can be eliminated in PF-QNM height images, which leads to a true height of the sample in the height image. In addition, by comparing Figure 2a and Figure 2e, we can see the RGO sheet was not damaged by the biased AFM tip. This is because the increase of adhesion caused by the applied tip bias is no more than 20 nN, which is about two orders of magnitude lower than the threshold force to destruct GO and RGO in our previous study [44]. Therefore, this method is not more destructive compared to the standard peak force mode without tip bias. This result shows that imaging in PF-QNM mode with a biased AFM tip can be used to simultaneously characterize topographic and dielectric properties under ambient conditions.

**Figure 3:**
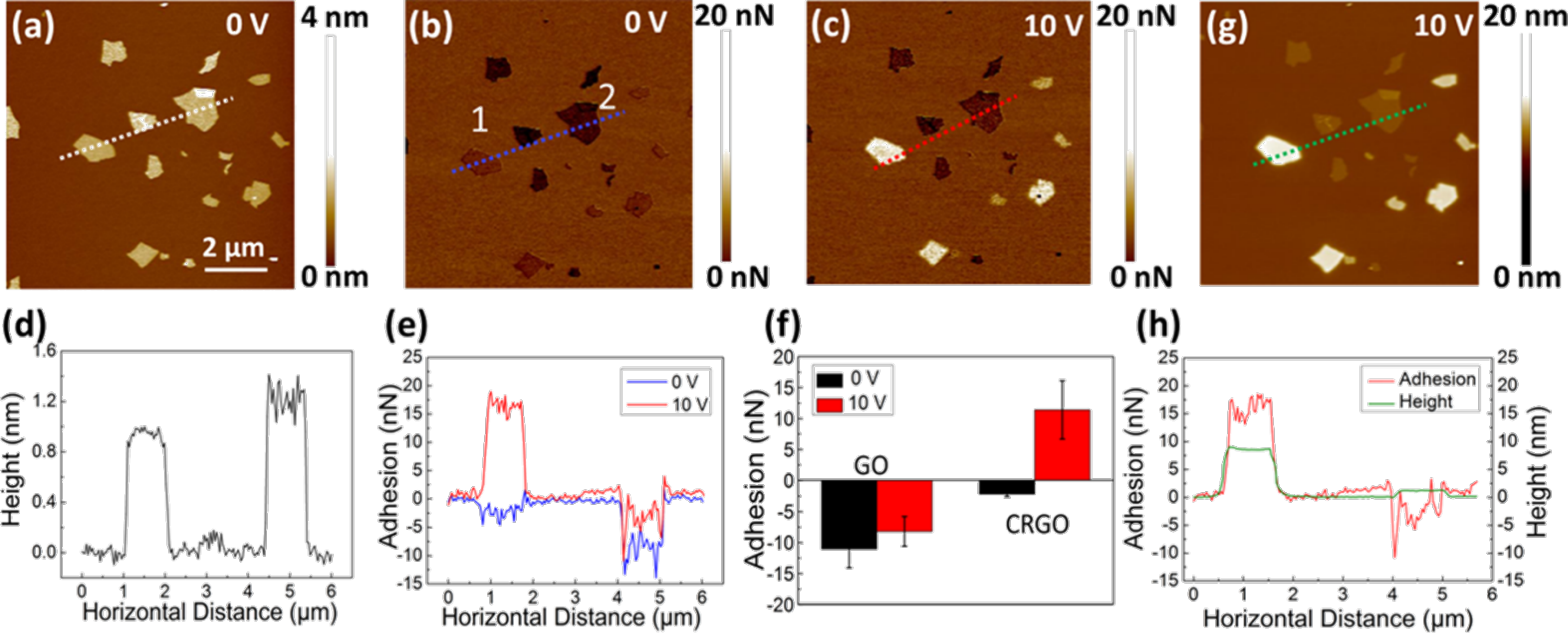
Discrimination of GO and CRGO on mica by adhesion mapping with a biased AFM tip. (a) Height and (b) adhesion force images taken simultaneously under an AFM tip bias of 0 V in PQ-QNM mode. (c) Adhesion force image taken under an AFM tip bias of 10 V. All of the images were taken with the peakforce set point at 5 nN under ambient conditions. (d) Cross-sectional profiles of the white dotted line in (a). (e) Cross sectional profiles of the blue and red dotted lines in (b) and (c), respectively. (f) The average adhesion forces of GO and RGO sheets under AFM tip biases of 0 and 10 V, respectively. (g) An in situ SPFM image of (c) taken under an AFM tip bias of 10 V with RH 10%. (h) Cross-sectional profiles of the red and green dotted lines in (c) and (g).

In addition, adhesion force mapping with a biased AFM tip can be used to distinguish between different dielectric materials at the nanoscale. In order to prove this, a mixed sample of GO and CRGO sheets was studied as a model system (Figure 3). CRGO is a product of GO after being chemically reduced by removing some oxygen-containing groups and has a similar thickness but a larger dielectric constant than GO [21,45]. Figure 3a shows a representative height image of a mixture of GO and CRGO on a mica substrate under the tip biased at 0 V under ambient conditions (room temperature 18–25 °C, RH 35–60%). All of the sheets in the height image have similar contrast (Figure 3a) but are quite different in the adhesion force images (Figure 3b,c). When the tip bias was 0 V, although the contrast of all sheets is darker than that of the mica substrate, the sheets can still be divided into two types, with one having a slightly smaller adhesion than the other (Figure 3b). However, we cannot infer which one has the larger dielectric constant from this image. When the tip bias increased to 10 V, the contrast of one type increased sharply and became much brighter than that of the mica substrate. The contrast for the other increased only slightly and remained darker than that of the mica substrate (Figure 3c). Two sheets in the centre of Figure 3b, which are marked as 1 and 2, were studied as representative of these two types. The cross-sectional profile (Figure 3d) reveals that the mean thicknesses of sheets 1 and 2 are 1 nm and 1.2 nm, respectively. Figure 3e indicates that the mean adhesion force of sheet 1 increased from −2.7 to 16.6 nN along with the increase in the tip bias from 0 to 10 V. In the meantime, the mean adhesion force of sheet 2 increased from −9.4 to −5.1 nN under the same conditions. The statistical average adhesion force from over 100 sheets in the mixed sample, the areas of which ranged from 0.01 to 4 μm^2^, showed that the increase in adhesion force was from −2.2 ± 0.6 nN to 12.8 ± 4.0 nN for type 1, and from −11.0 ± 2.9 nN to −8.3 ± 2.3 nN for type 2 (Figure 3f). According to Equation 1, the increase in the adhesion force caused by the external voltage, which is rooted in the polarization of the sample, is positively related to the dielectric constant of the sample. Therefore, type 1, which displayed a larger increase in adhesion force, is CRGO. That is, GO and CRGO in the mixed sample can be distinguished clearly by this method. It is worth noting that the contact potential differences between the AFM tip and GO/RGO are about three orders of magnitude lower than the tip bias in adhesion mapping (Supporting Information File 1, Figure S1). So the effect of the contact potential difference between the tip and our sample was ignored in our experiments.

A comparison study of dielectric property mapping by adhesion force and SPFM was also carried out. Figure 3g shows an in situ SPFM image of Figure 3c taken with an AFM tip biased at 10 V at RH 10% [32]. All of the sheets with increased adhesion forces in Figure 3c have increased apparent heights in the corresponding SPFM image, and the apparent heights of the other sheets remain unchanged. That is, adhesion mapping under a biased AFM tip in PF-QNM mode is in good agreement with SPFM imaging in local dielectric property detection. Figure 3h shows the cross-sectional profiles along the red and green dotted lines in Figure 3c and Figure 3g, respectively. It indicates that the surfaces of the sheets in SPFM images are quite smooth in comparison with those in the adhesion image. The small grainy structures with a lateral size of less than dozens of nanometres on the basal planes of GO and RGO sheets, which are regarded as oxygen-containing functional groups [21,31], cannot be observed in the SPFM image but can be seen in the adhesion image. This is because SPFM works in the long-range electrostatic interaction region, but the adhesion mapping in PF-QNM mode works on the sample surface all the time, no matter if the AFM tip is biased or not. This result proves that adhesion force mapping under a biased AFM tip has the same capacity as SPFM to distinguish local dielectric distribution, but has a higher lateral resolution comparable to the conventional AFM modes.

**Figure 4:**
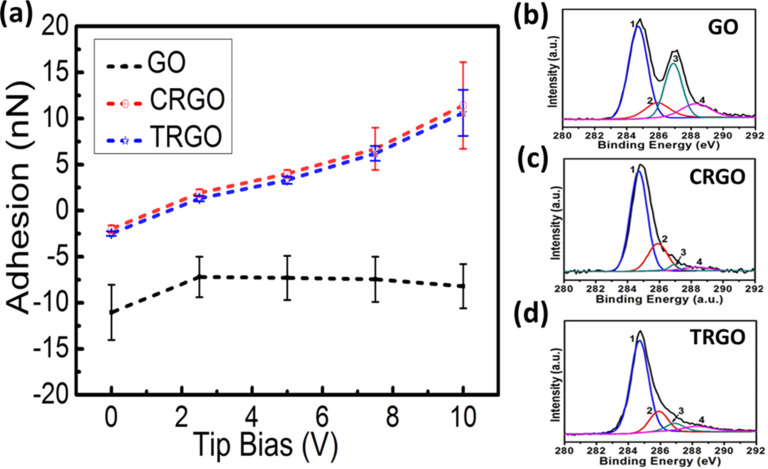
The dependence of the adhesion force on the AFM tip bias for three types of single-layered GO and RGO. (a) The average adhesion forces of the GO, CRGO (chemically reduced by saturated hydrazine vapor at 80 °C for 1 h) and TRGO (thermally reduced at 180 °C for 15 min) plotted against different biases of the AFM tip. XPS spectra of (b) GO, (c) CRGO and (d) TRGO. The peaks 1, 2, 3, and 4 in the coloured curves correspond to C=C/C–C in aromatic rings, C–O (epoxy and alkoxy), C=O, and COOH groups, respectively. The C/O ratios for these samples are 1:1, 3.3:1, and 3.2:1, respectively.

The dependence of the adhesion force under a biased AFM tip on the reduction degree of GO was also studied through X-ray photoelectron spectroscopy (XPS) experiments. Figure 4a shows the average adhesion forces of the three samples plotted against different biases of the AFM tip. For GO, the mean value of the adhesion force initially increased from −11.0 ± 2.9 nN to −7.2 ± 2.2 nN when the tip bias increased from 0 V to 2.5 V, and then decreased slightly to −8.3 ± 2.3 nN along with the tip bias rising to 10 V. For CRGO and thermally reduced GO (TRGO), the initial values of the adhesion force with a tip bias of 0 V were −2.2 ± 0.4 nN and −2.3 ± 0.3 nN, respectively. The values subsequently increased monotonically with almost exactly the same trend to 11.2 ± 4.7 nN and 11.0 ± 2.5 nN until the tip bias reached 10 V. Since all of the adhesion forces mentioned in this paper are relative values to mica, the effect of system drift on force–distance curves during the imaging process can be eliminated (Supporting Information File 1, Figure S2). The increases in the adhesion forces of CRGO and TRGO when the tip bias increased from 0 to 10 V are 13.4 nN and 13.3 nN, respectively, which are very similar and almost five times larger than that of GO. Figure 4b–d shows XPS spectra of single-layered GO, CRGO, and TRGO, respectively, which reveal that the C/O ratios of GO increased from 1:1 to 3.3:1 and 3.2:1 after being chemically and thermally reduced, respectively. In general, the reduction degree of GO is positively related to its dielectric properties [21,46]. This result further confirms that the reduction degree of GO is positively related to the adhesion force caused by the biased AFM tip.

## Conclusion

In summary, it was found that the adhesion force between a dielectric sample and a biased AFM tip was affected by sample polarization. The increase in the adhesion force caused by an external voltage is positively related to the dielectric properties of the sample. Based on this principle, GO and its reduction products can be precisely distinguished by adhesion mapping using a biased AFM tip. This experiment, in principle, proves that imaging in the PF-QNM mode with a biased AFM tip can be used to simultaneously characterize topographic and dielectric properties in the nanoscale under ambient conditions with a high lateral resolution that is comparable to the conventional AFM modes. This method provides a general but simple approach for the fast characterization of the local dielectric properties of graphene-based materials and will facilitate their future applications in the energy generation and storage devices.

## Experimental

### Sample preparation

An aqueous solution of single-layered GO sheets was prepared from graphite powder following a modified Hummer’s method [47–49]. A drop of 10 µL of as-prepared GO solution (50 ng/µL) was placed onto a mica substrate. Chemical reduction of GO was performed by exposure to a saturated vapour of hydrazine monohydrate (85 wt % in water, Sinopharm) in a sealed Petri dish at 80 °C for 1 h. Thermal reduction of GO was carried out in a vacuum oven at 180 °C for 15 min. A hybrid GO/RGO sample was made by depositing another drop of GO solution onto the substrate on which reduced GO had been deposited.

### Characterization

The samples were characterized by using a MultiMode 8 AFM (Bruker) equipped with a J scanner. Silicon cantilevers coated with a 30 nm Pt layer with a nominal spring constant of 2.8 N·m^−1^ and oscillating frequencies of 60–90 kHz (NSC18/ Pt, MikroMasch Co.) were used. Height and adhesion mapping were conducted in PeakForce Quantitative Nano-Mechanics (PF-QNM) mode, in which the maximum force (peak force) applied to the sample by the tip was directly regulated through the peak force setpoint and kept constant throughout the whole scan. In this mode, the peak force amplitude was set at 150 nm, the *Z*-piezo oscillation frequency at 2.0 kHz, and the scan rate at 1 Hz. Voltage to the tip was applied using the scan parameter “tip bias”. All AFM experiments were conducted under ambient conditions at a room temperature of 18–25 °C and relative humidity of 35–60%. AFM images of the samples were processed using the software Nanoscope Analysis v1.7. For each image, a first-order flatten correction was applied to remove sample inclination. The reduction extent of the GO was characterized by X-ray photoelectron spectroscopy (XPS, AXIS Ultra DLD, Kratos).

## Supporting Information

File 1 Additional experimental data.
