# Supplementary material for: Nanoscale mapping of dielectric properties based on surface adhesion force measurements
Source: Beilstein J Nanotechnol. 2018 Mar 16;9:900–6. doi: 10.3762/bjnano.9.84 (PMC5870145; doi:10.3762/bjnano.9.84)
Supplement: File 1 — Additional experimental data. [file Beilstein_J_Nanotechnol-09-900-s001.pdf]

Supporting Information

for

Nanoscale mapping of dielectric properties based on surface adhesion force measurements

Ying Wang[1], Yue Shen[2], Xingya Wang[1,3], Zhiwei Shen[1,3], Bin Li[1], Jun Hu[1] and Yi Zhang*[1]

Address: [1]Key Laboratory of Interfacial Physics and Technology and Laboratory of Physical Biology, Shanghai Institute of Applied Physics, Chinese Academy of Sciences, Shanghai 201800, China; [2]Key Laboratory of Salt Lake Resources Chemistry of Qinghai Province, Qinghai Institute of Salt Lakes, Chinese Academy of Sciences, Xining, Qinghai 810008, China and [3]University of Chinese Academy of Sciences, Beijing 100049, China

Email: Yi Zhang* - zhangyi@sinap.ac.cn

* Corresponding author

Additional experimental data

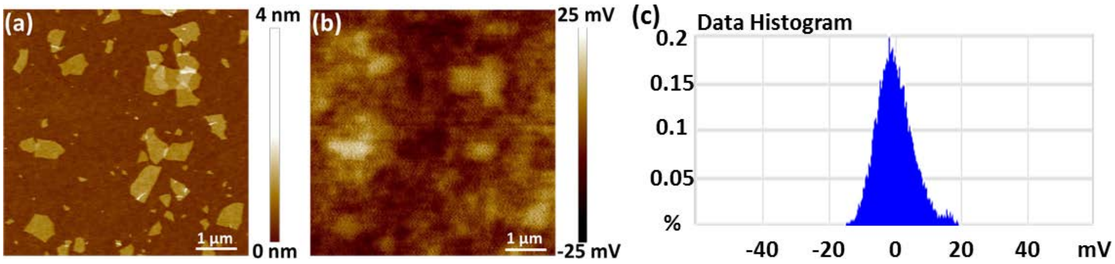

Figure S1: The contact potential difference between the tip and the sample. (a) Height image of GO/RGO hybrid sample in KPFM mode. (b) The corresponding surface potential image to (a). (c) The distribution of surface potential in (b). The measured contact potential difference between the AFM tip and GO/RGO in KPFM mode is between −16 mV and 19 mV.

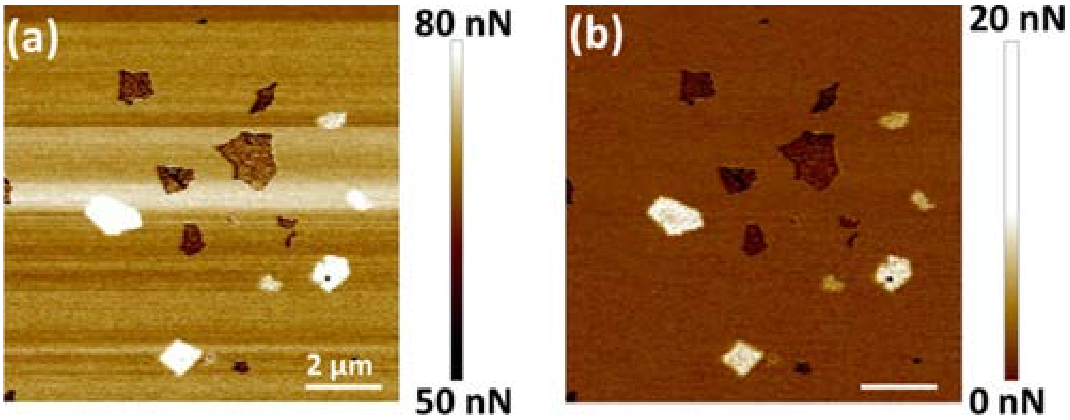

Figure S2: System drift of the raw adhesion image. (a) Raw adhesion image of hybrid GO and rGO sample. (b) Adhesion image after first-order flatten correction of (a).
